# Protocol for a Phase 1, Open-Label, Multiple-Center, Dose-Escalation Study to Evaluate the Safety and Tolerability of ADR-001 in the Treatment of Immunoglobulin A Nephropathy

**DOI:** 10.3389/fmed.2022.883168

**Published:** 2022-05-27

**Authors:** Akihito Tanaka, Kazuhiro Furuhashi, Kumiko Fujieda, Kayaho Maeda, Shoji Saito, Tetsushi Mimura, Yosuke Saka, Tomohiko Naruse, Takuji Ishimoto, Tomoki Kosugi, Fumie Kinoshita, Yachiyo Kuwatsuka, Shinobu Shimizu, Yasuhiro Nakai, Shoichi Maruyama

**Affiliations:** ^1^Department of Nephrology, Nagoya University Hospital, Nagoya, Japan; ^2^Department of Nephrology, Kasugai Municipal Hospital, Kasugai, Japan; ^3^Department of Nephrology and Rheumatology, Aichi Medical University, Nagakute, Japan; ^4^Department of Nephrology, Nagoya University Graduate School of Medicine, Nagoya, Japan; ^5^Department of Advanced Medicine, Nagoya University Hospital, Nagoya, Japan

**Keywords:** IgA nephropathy, mesenchymal stem cell, MSC, adipose-derived mesenchymal stem cell, ASC

## Abstract

**Introduction:**

Immunoglobulin A (IgA) nephropathy is a disease that presents with urinary symptoms such as glomerular hematuria and urinary protein positivity, with predominant deposition of IgA in the mesangial region of the glomerulus. Corticosteroids are mainly used for treatment; however, infection is a serious adverse event, and evidence regarding therapeutic efficacy is insufficient, thus new treatments are strongly desired. Mesenchymal stem cells (MSCs) contribute to the amelioration of inflammation and recovery of organ function in inflammatory environments by converting the character of leukocytes from inflammatory to anti-inflammatory and inducing the proliferation and differentiation of organ component cells, respectively. These properties of MSCs have led to their clinical application in various inflammatory diseases, but this study is the first clinical trial of MSCs for refractory glomerulonephritis in the world. This study is registered and assigned the number, jRCT2043200002 and NCT04342325.

**Methods:**

This will be a phase 1, open-label, multiple-center, dose-escalation study of adult patients with refractory IgA nephropathy resistant to or difficult to treat with existing therapies. ADR-001 will be administered intravenously to from three to six patients at a dose of 1 × 10^8^ cells once in the first cohort and to six patients twice at 2-week intervals in the second cohort, and observation will continue until 52 weeks. The primary endpoint will be the evaluation of adverse events up to 6 weeks after the start of ADR-001 administration. Secondary endpoints will be the respective percentages of patients with adverse events, clinical remission, partial remission, remission of urine protein, remission of hematuria, time to remission, changes in urine protein, hematuria, and estimated glomerular filtration rate.

**Results:**

Following the administration of ADR-001 to patients with IgA nephropathy, the respective percentages of patients with adverse events, asymptomatic pulmonary emboli, clinical remission, partial remission, urine protein remission, hematuria remission, their time to remission, changes in urine protein, hematuria, and glomerular filtration rate will be determined.

**Conclusion:**

This study will evaluate the safety and tolerability of ADR-001 and confirm its therapeutic efficacy in adult patients with refractory IgA nephropathy.

## Introduction

Immunoglobulin A (IgA) nephropathy presents with urinary findings suggestive of nephritis, such as glomerular hematuria and positive urinary protein. It is characterized by predominant IgA deposition in the glomeruli.

Many cases are detected as asymptomatic urine analysis abnormalities; however, in some cases, the kidney damage progresses without a noticeable onset of the disease, with the disorder being detected by a hypertension diagnosis or blood test abnormalities such as blood urea nitrogen (BUN) and serum creatinine levels. In addition, although rare, edema due to acute nephritis or nephrotic syndrome may trigger detection, including gross hematuria associated with acute upper respiratory infection, mainly tonsillitis or pharyngitis. For a definitive diagnosis, it is necessary to show that IgA is deposited mainly in the mesangial region of the glomeruli by renal biopsy. Currently, adult patients with IgA nephropathy are treated with blood pressure management, lifestyle modification, and maximally tolerated dose of renin-angiotensin system (RAS) inhibitors. Further, corticosteroids and immunosuppressive drugs are used for patients at high risk of progressive kidney disease despite the above treatments. In some Asian countries, palatine tonsillectomy (plus steroid pulse therapy), antiplatelet drugs, and n-3 fatty acids (fish oils) are also used (1). Treatment with corticosteroids is the gold standard for IgA nephropathy with high risk of disease progression, but there is no established treatment regimen for patients who are refractory to steroid therapy. In particular, in Japan, tonsillectomy is performed together with steroid therapy to enhance the therapeutic effect; however, the level of evidence to support this is currently not significant. Recently, two clinical trials have been reported (2, 3). First, as for the STOP-IgA trial, it failed to demonstrate statistical efficacy in preventing the progression of kidney dysfunction and showed high risk of severe infectious disease (2). Second, as for the TESTING trial, steroid therapy showed statistical efficacy in preventing the progression of kidney dysfunction, but adverse events such as infections led to early discontinuation of the trial (3). Therefore, there is a critical need for immunosuppressive therapy for treatment-resistant IgA nephropathy that does not increase side effects. Owing to this, we hypothesized mesenchymal stem cells (MSCs) as a new treatment approach because of their therapeutic properties.

Mesenchymal stem cells have shown beneficial effects in a broad range of animal disease models by promoting regeneration and/or enhancing immunoregulatory effects. Cell therapy for kidney disease using MSCs began with a report of bone marrow-derived mesenchymal stem cell (BM-MSC) administration in a mouse model of cisplatin nephropathy (4). Since then, a number of studies have reported the therapeutic effects of BM-MSCs in acute kidney injury (AKI), nephritis, and chronic kidney disease (CKD) models, demonstrating that MSCs have a renal protective effect. MSCs have been clinically applied to various diseases, especially those that are difficult to treat with currently existing immunosuppressive drugs. Based on our research findings, we recognized the therapeutic properties that could be harnessed from MSCs, viz. strong anti-inflammation properties and their ability not to induce harmful immunosuppression even in excessive amounts. In addition to the previously mentioned immunosuppressive effects, MSCs have a negative HLA-DR and are not prone to rejection, making it possible to perform an allotransplant in which someone else’s MSCs are administered even when the blood type or HLA is incompatible. For these reasons, MSCs have been tested in clinical trials for a variety of inflammatory diseases, including renal disorders (5). Adipose-derived mesenchymal stem cells (ASCs) have a higher regenerative capacity than BM-MSCs because they secrete more growth factors such as hepatocyte growth factor and vascular endothelial growth factor. They also secrete more immunomodulatory factors and have been reported to have a high immunomodulatory function (6, 7). Furthermore, the physical and mental hurdles for donors are low because fat is relatively safe and easy to harvest. In culture, ASCs have excellent proliferative capacity and efficient culture techniques have been established, making it is possible attain the number of cells required for therapy in a relatively short period of time. Therefore, ASCs have many advantages over other cells and are considered to have high feasibility and therapeutic efficacy in clinical applications.

Adipose-derived mesenchymal stem cells have been shown to reduce serum creatinine concentration, BUN, and kidney weight in a rat model of anti-glomerular basement membrane nephritis in non-clinical studies (7). On the other hand, there is no appropriate animal model of IgA nephropathy. However, from the pathogenesis of IgA nephropathy (J Am Soc Nephrol. 2011 Oct;22 (10):1795-803.) and previous reports that MSCs are effective not only in cellular immunity but also in humoral immunity, such as suppression of antibody production (7–9), we believe that MSCs may be effective in IgA nephropathy. Considering clinical application, more than 1,000 clinical trials using MSCs have been conducted worldwide (5), and more than 30 clinical trials have been conducted in the field of nephrology transplantation, CKD, AKI, diabetic kidney disease, and focal segmental glomerulosclerosis. Some promising results have been observed, such as the administration of ASCs for renal artery stenosis (10). However, clinical studies of ASCs in CKD have been limited to case reports, and therapeutic potentials of administered allogeneic ASCs in patients with therapy-resistant nephritis have not been clearly reported (11). Therefore, it is eagerly awaited to clarify this. In this study, we will conduct a prospective clinical trial to evaluate the safety and tolerability of one and two intravenous doses of ADR-001, allogeneic ASCs, as a primary endpoint and to confirm therapeutic efficacy as a secondary endpoint in patients with refractory IgA nephropathy who are resistant to or difficult to manage with existing therapies.

## Method and Design

### Design

**FIGURE 1:**
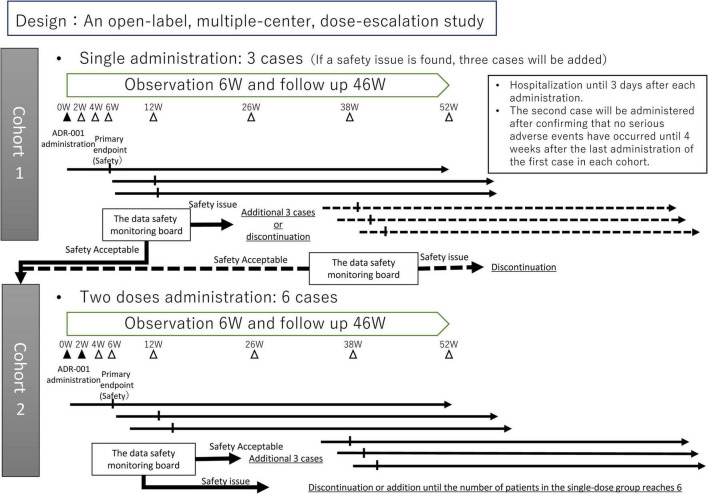
Outline of the study. The study will start with cohort 1, and after assessment, then move to cohort 2.

This will be an open-label, multiple-center, dose-escalation study of adult patients with refractory IgA nephropathy who are resistant to or difficult to manage with existing therapies. Figure 1 shows an outline of the study. ADR-001 will be administered intravenously at a dose of 1 × 10^8^ cells once in the first cohort and twice at 2-week intervals in the second cohort, and evaluation and observation will continue until 52 weeks. Supplementary Figure 1 presents the schedule of the study. To conduct a safe trial, the protocol was designed so that the first cohort of single-dose patients will be completed before proceeding to the second cohort of double-dose patients. A maximum of 12 subjects will be enrolled from the Nagoya University Hospital and Kasugai Municipal Hospital. The main analysis population will be the safety analysis population for the analysis of the primary endpoint and the largest analysis population full analysis set (FAS) for the analysis of the secondary endpoint. Analysis will also be conducted on the analysis population conforming to the study protocol Per Protocol Set (PPS) to confirm the robustness of the analysis results. The trial will be conducted in compliance with the Declaration of Helsinki and Japan’s Ministerial Ordinance on Good Clinical Practice for Cellular and Tissu-based Products.

### Setting

This study will be conducted at the Nagoya University Hospital and Kasugai Municipal Hospital in Aichi, Japan.

### Population

The target population will be adult patients with refractory IgA nephropathy who are resistant to or difficult to treat with existing therapies. Consent will be obtained from all patients prior to commencing the study.

### Eligibility Criteria

At the time of enrollment, patients who meet Inclusion Criteria 1–4 and do not conflict with any of Exclusion Criteria 1–15 will be included. However, in the first cohort, only 2–1 of Inclusion Criteria 2 will be applied, and in the second cohort, 2–1, 2–2, and 2–3 will be applied. The estimated glomerular filtration rate (eGFR) will be calculated using the CKD-EPI formula (12).

### Inclusion Criteria

1. Patients diagnosed with IgA nephropathy following renal biopsy.
2. Patients who meet any of the following criteria. We targeted IgA nephropathy with persisitent proteinuria.

2-1. Patients with urine protein >0.5 g/gCr and eGFR >60 mL/min/1.73 m^2^ at screening, even if corticosteroids were used for >6 months before screening.
2-2. Patients with urine protein >1.0 g/gCr and eGFR >30 mL/min/1.73 m^2^ at screening, even if corticosteroids were used for >6 months before screening.
2-3. Patients with urine protein between 0.5 g/gCr and 1.0 g/gCr and eGFR between 20 mL/min/1.73 m^2^ and 60 mL/min/1.73 m^2^ at screening, or those with urine protein >1.0 g/gCr and eGFR between 20 mL/min/1.73 m^2^ and 30 mL/min/1.73 m^2^ at screening.

3. Patients over 20 years old.
4. Patients who have given their written consent after receiving a full explanation of the clinical trial.

### Exclusion Criteria

1. Patients with nephropathy other than IgA nephropathy (membranous nephropathy, microvariant nephrotic syndrome, focal segmental glomerulosclerosis, etc.), and patients with primary and secondary nephrotic syndrome (caused by IgA nephropathy or autoimmune diseases, metabolic diseases, infections, allergies, hypersensitive diseases, tumors, drugs, etc.).
2. Patients who started or increased the dose of medication for IgA nephropathy within 3 months prior to consent requisition, or who underwent palatine tonsillectomy within 6 months prior to consent requisition.
3. Patients who have had other cell-based therapies.
4. Patients who participated or were currently participating in other clinical trials within 3 months prior to consent requisition. However, if the study was an observational study within the scope of the approved indication, dosage, and administration, inclusion is acceptable.
5. Patients who have received a kidney transplantation or are scheduled to receive a kidney transplantation within the next 3 years.
6. Patients with poorly controlled diabetes mellitus (HbA1c > 7.5%).
7. Patients with malignant neoplasms, patients who have had malignant neoplasms within 5 years prior to consent requisition, or patients who were thought to have malignant tumors based on various tests. However, patients with no recurrence or metastasis during 5 years of follow-up after primary treatment (colectomy) will be included.
8. Patients who have or are suspected to have active infections that require treatment with antibacterial, antifungal, or antiviral drugs intended to act systemically at the time of obtaining consent.
9. Patients who are positive for hepatitis B surface (HBs) antigen, HBs antibody, hepatitis B core (HBc) antibody, hepatitis C virus antibody, human immunodeficiency virus antibody, human T-cell lymphotropic virus type 1 antibody, or syphilis serum reaction. Patients with positive HBs antibody or HBc antibody can be registered only if hepatitis B virus DNA quantification is negative or below detection sensitivity.
10. Patients with a history of serious hypersensitivity or anaphylactic reactions.
11. Patients who are allergic or have a history of allergy to penicillin antibiotics, aminoglycoside antibiotics, or dimethyl sulfoxide.
12. Patients with serious complications not related to IgA nephropathy (liver disease, renal disease, cardiac disease, pulmonary disease, hematological disease, brain disease, etc.).
13. Patients who are likely to bleed, patients who have recently undergone central nervous system surgery or trauma, patients who have a history of hypersensitivity to heparin, and patients who have a history of heparin-induced thrombocytopenia.
14. Female patients who are pregnant, lactating, or may be pregnant, or patients of either sex who are unable to consent to contraception under the guidance of the investigator during the study period.
15. Other patients who are judged to be inappropriate by the study investigator.

### Screening Process

After obtaining consent, the following tests and observations will be performed or confirmed before registration:

1. Confirmation of subject eligibility based on inclusion/exclusion criteria.
2. Background check on the subject (date of birth, sex, weight, and height).
3. Status of primary disease (duration of IgA nephropathy and historical eGFR) and previous treatment (type and duration of drug therapy, lifestyle, and dietary guidance for IgA nephropathy).
4. Confirmation of medical history and complications (clinically significant diseases within the past 6 months).
5. Vital signs (systolic blood pressure, diastolic blood pressure, heart rate, and body temperature).
6. Oxygen saturation.
7. Clinical examination.
8. Check for concomitant medications and concomitant therapy.
9. Simple CT scan of the area between the chest and pelvic region.
10. Echocardiography and electrocardiography.

### Regulations on Concomitant Medications and Therapy

#### Concomitantly Prohibited Drugs and Therapies

The following medical interventions or administration of the following therapies or drugs will not be acceptable from the time of giving consent and throughout the study period:

1. Tonsillectomy.
2. Kidney transplantation.
3. Other cell-based therapies.
4. Other investigational drugs.

#### Concomitant Use of Drugs and Therapies

Consent should be obtained after confirming that the administration of therapies and drugs used for systemic effects will not be changed in principle from 3 months prior to the time of consent until the start of the trial-drug administration. All drugs and therapies which may affect the level of proteinuria will not be permitted to be initiated or increased from the start until the end of the study. However, temporary administration of the following agents for the treatment or prevention of adverse events will be acceptable:

1. Corticosteroids (prednisolone, methylprednisolone).
2. Immunosuppressive drugs (cyclophosphamide, azathioprine, cyclosporine, mycophenolate mofetil, and mizoribine).
3. Renin-angiotensin system inhibitors.
4. Antiplatelet drugs (dipyridamole, dilazep hydrochloride, ticlopidine, and aspirin) and anticoagulants (warfarin).
5. n-3 fatty acids (fish oil, eicosapentaenoic acid, and omega-3 fatty acid).

#### Administration of ADR-001

ADR-001 is provided by ROHTO Pharmaceutical Co., Ltd. Stromal vascular fraction is prepared from obtained adipose tissue and MSCs are extracted. Each dose of 1 × 10^8^ ADR-001 will be administered intravenously. The first cohort will receive one dose, and the second cohort will receive two doses with a 2-week interval.

1. Method of preparation

ADR-001 will be melted in a constant temperature water bath at 37°C, and then diluted at the time of use.

2. Premedication

To prevent hypercoagulability that may occur during administration, a pre-dose of 1,000 U of heparin will be administered approximately 5 min prior to administration of ADR-001. Simultaneously, 500 U of heparin per hour will be administered to coincide with the time of administration of ADR-001.

3. Measurement of vital signs

Within 10 min prior to the administration of ADR-001, body temperature, blood pressure, pulse rate, and oxygen saturation will be measured. During administration, the pulse oximeter will be kept on to continuously measure oxygen saturation. The necessary tools and medicines for first aid will be prepared and immediate action will be taken if a serious anaphylactic reaction occurs.

#### Study Endpoints

The primary endpoint will be the evaluation of adverse events up to 6 weeks after the start of ADR-001 administration by interview, visual examination, palpation, and clinical examination, such as electrocardiogram, cardiac ultrasonography, computed tomography, and blood tests. Secondary endpoints will be the respective percentages of patients with adverse events, clinical remission, partial remission, remission of urine protein, remission of hematuria, time to remission, changes in urine protein, hematuria, and eGFR. Each index will be measured at 2, 4, 6, 12, 26, 38, and 52 weeks post-dose, and remission will be considered when the criteria are met at least three times in a row. Safety assessment will be based on adverse events, vital signs, and laboratory data.

#### Sample Size

The target number of patients will be three to six in the first cohort and six in the second cohort, for a total of 9–12.

#### Clinical Trial Period

Clinical trials will be conducted from April 2020 to March 2023. Cases will be enrolled from April 2020 to September 2021. The duration of the clinical trial for each subject will be approximately 56 weeks (4 weeks for the screening period and 52 weeks for the observation period).

#### Data Analysis Plan

The main analysis population will be the safety analysis population for the analysis of the primary endpoint and the largest analysis population (FAS) for the analysis of the secondary endpoint. Analysis will also be conducted on the analysis population conforming to this study protocol (PPS) to confirm the robustness of the analysis results. Adverse events, which are the primary and safety endpoints, will be classified and aggregated using CTCAE v5.0/MedDRA/J v22.1, and incidence rates and 95% confidence intervals will be calculated. In addition, the incidence rates will be calculated for each of the following categories: cohort, organ major classification, preferred term, severity, and association with ADR-001. As the secondary endpoint, we will calculate the percentage and 95% confidence interval and estimate each of the following parameters using the Kaplan–Meier method: patients who achieve clinical remission, partial remission, urinary protein remission, hematuria remission, and time to remission. Summary statistics (mean, standard deviation, etc.) will be calculated for urine protein, hematuria, and eGFR. For safety assessment endpoints such as vital signs and clinical laboratory values, summary statistics (mean, standard deviation, etc.) will be calculated, and their transition will be shown.

#### Ethical Conduct of the Study

This clinical trial will be conducted in compliance with the Declaration of Helsinki and Japan’s Ministerial Ordinance on Good Clinical Practice for Drugs. The trial has been registered in the Japan Registry of Clinical Trials; Clinical Research Protocol Number: jRCT2043200002 and ClinicalTrials.gov Identifier: NCT04342325. Prior to the start of the clinical trial, all subjects will be fully informed using an IRB-approved written explanation (Approval number at Nagoya University: 312008 and at Kasugai Municipal Hospital: CAMCR-013), and informed consent will be obtained from all participants.

#### The Data and Safety Monitoring Board

The data and safety monitoring board (DSMB) consists of three specialists in nephrology independent from the trial investigators. The DSMB will be held at predefined times in both cohorts, total four times: at 6 weeks after administering to the first case and 6 weeks after administering to the third case in each cohort. When a product-related severe adverse event occurs or when investigators consider that it should be convened due to safety concerns, the council will also be held. The DSMB will recommend whether this trial should be moved forward or be discontinued (Figure 1).

#### Criteria for Discontinuation

(1) When a patient declines to continue participation in the clinical trial or withdraws consent.
(2) When it becomes impossible to continue the clinical trial due to transfer to a different hospital, or change of address during the course of the clinical trial.
(3) When the patient is found to be ineligible after enrollment.
(4) When it is difficult to continue the clinical trial due to exacerbation of complications.
(5) When it is difficult to continue the clinical trial due to adverse events.
(6) When pregnancy of the patient is confirmed.
(7) When the entire clinical trial is terminated.
(8) When the “restricted drugs and therapies” is administered.
(9) When the investigator determines that it is appropriate to discontinue the clinical trial for other reasons.

## Discussion

Herein, we report a protocol for the world’s first clinical trial of allogeneic ASCs for refractory IgA nephropathy. The purpose of this study is to evaluate the safety and tolerability of ADR-001 MSCs derived from allogeneic adipose tissue in patients with IgA nephropathy. IgA nephropathy is a disease with a poor prognosis, with 40% of patients developing end-stage renal failure within 20 years after renal biopsy (13, 14). Although there is no established treatment for refractory IgA nephropathy, immunosuppressive drugs are widely used. However, the evidence for their use is controversial. For example, it has been reported that the addition of immunosuppressive therapy such as cyclophosphamide and azathioprine to active supportive therapy in patients with high-risk IgA nephropathy did not significantly improve outcomes (2). Patients who received immunosuppressive drugs during the 3-year study period also experienced more side effects, and the rate of eGFR decline was unchanged (2). Most of the randomized controlled trials that have demonstrated the efficacy of corticosteroids in reducing renal function and improving proteinuria in IgA nephropathy have been based on patients with CKD stage G1–2 IgA nephropathy with urinary protein >1.0 g/day (15, 16). On the other hand, there are only a few studies on patients with IgA nephropathy whose urinary protein is <1 g/day and whose renal function is < CKD stage G3 (3), which is insufficient to investigate the therapeutic effect of corticosteroids. For these reasons, there is a strong need to establish a new treatment protocol to improve the prognosis of IgA nephropathy. Consequently, we decided to investigate the use of MSCs as a novel candidate for the treatment of IgA nephropathy.

This will be the first clinical trial of MSCs in patients with refractory IgA nephropathy, and positive therapeutic outcomes are expected. In animal experiments, MSCs were shown to be effective against highly inflammatory nephritis (7). After MSCs are administered, they enter the bloodstream and secrete various inflammation-regulating cytokines to control local inflammation when they reach the local inflammatory area. Therefore, while inflammation is locally controlled, systemic side effects are unlikely to occur. In other words, MSCs are expected to have improved therapeutic effects and limited side effects, such as infections caused by steroids and immunosuppressive drugs. Our study also suggests that MSCs do not cause excessive immunosuppression (17). However, a possible side effect is that the cell mass itself and a subsequent blood clot can induce pulmonary embolism. We are planning to prevent potential pulmonary embolism as much as possible by using heparin in combination with ADR-001. Although bleeding tendency needs to be taken into account, we set a clinically acceptable heparin dose based on the one used during extracorporeal circulation for hemodialysis. Concerns about pulmonary embolism also increase with the number of cells administered; however, in our trial, the number of cells administered will be based on past administration for other diseases and will not particularly be high. In addition, ECG and SpO_2_ monitors will be worn during administration, and ECG, laboratory tests, such as d-dimer, and echocardiography will be performed on the day of administration and the following day to detect early signs of right ventricular strain and pulmonary embolism. The transplantation of induced pluripotent stem cells, a primitive stem cell, has raised concerns about the occurrence of teratomas in animal experiments. Since MSCs do not proliferate indefinitely, there is little concern about the development of malignant tumors; however, to guarantee safety, CT scans will be performed to check for malignant tumors of the whole body at 52 weeks. Furthermore, patients who present with concerns regarding malignancy prior to the administration of ADR-001 will not be included in the study. The number of doses will be one for the first cohort and two for the second cohort. Based on our results from animal models, we expect that the more times ADR-001 is administered, the more effective the treatment will be. However, since the main purpose of this study is to confirm safety, and since this will be the world’s first study on nephritis, we decided to prioritize safety and start the study with a single dose. In addition, the first cohort will include a population with good renal function for safety reasons. The second cohort will include patients with a wide range of renal functions. Based on the results of this study, we will determine the level of renal function that is best suited for treatment with ADR-001 in terms of safety and efficacy.

This study protocol has some limitations. First, the dose increase of steroids and RAS inhibitors is restricted, so we cannot conclude on the effect of the combination of these drugs. Second, the safety and efficacy of the drug in patients with renal function too impaired to enter the study cannot be determined.

## Dissemination

The findings of this study will be disseminated *via* international peer-reviewed journals and scientific conferences.

## Ethics Statement

The studies involving human participants were reviewed and approved by Nagoya University Hospital Institutional review board (IRB), Nagoya University. The patients/participants provided their written informed consent to participate in this study.

## Author Contributions

KuF and AT mainly wrote the manuscript. All authors conceptualized the research idea and the study design, however, the contribution of KaF was particularly significant. AT, KaF, KuF, KM, SSa, TM, YS, TN, TI, TK, and SM recruited and treated patients. FK mainly performed the data analysis. FK, YK, SSh, and YN performed the data management. All authors performed data interpretation, contributed with important intellectual content during the writing of the manuscript and its revisions. Thus, each author is personally accountable for their contributions. Additionally, all authors agree to ensure that questions pertaining to the accuracy and integrity of any portion of the work will be appropriately investigated and resolved.

## Conflict of Interest

The authors declare a potential conflict of interest and state it below: We received research funding and the investigational product ADR-001 from ROHTO Pharmaceutical Co., Ltd.

## Publisher’s Note

All claims expressed in this article are solely those of the authors and do not necessarily represent those of their affiliated organizations, or those of the publisher, the editors and the reviewers. Any product that may be evaluated in this article, or claim that may be made by its manufacturer, is not guaranteed or endorsed by the publisher.

## Supplementary Material

Supplementary Figure 1 **(A)** Overall schedule of the study. IC, informed consent; CT, computed tomography; UCG, ultrasound cardiography; ECG, electrocardiogram; SpO_2_, percutaneous oxygen saturation; and HbA1c, hemoglobin A1c. 1: If necessary, hospitalization a few days prior to administration and continued hospitalization beyond 2 days after administration is acceptable. 2: If the results are within the acceptable range and it is judged that there is no need to test again, the results can be used as the results at the time of discontinuation. However, from the viewpoint of safety, test items that are applicable to the test at the time of discontinuation and that are specified at each evaluation point should be performed at the specified evaluation point as much as possible. 3: Available as a result of screening if data is available within 28 days prior to obtaining consent. 4: Available as a result of screening if data is available within 90 days prior to registration. 5: Only women with childbearing potential are eligible. A woman with childbearing potential is defined as a woman who does not fall into any of the following categories: (1) Women who have not had a menstrual period for at least 1 year; (2) Women who underwent hysterectomy; and (3) Women who underwent bilateral oophorectomy. 6: Conducted prior to the administration of ADR-001 on the day before or on the day of administration. 7: Collect between 2 days prior to dosing and administration of ADR-001. 8: Discharge after confirming the coagulation test results. **(B)** Schedule of the study for each cohort at 2 weeks after first administration. IC, informed consent; CT, computed tomography; UCG, ultrasound cardiography; ECG, electrocardiogram; SpO_2_, percutaneous oxygen saturation; and HbA1c, hemoglobin A1c. 1: If necessary, hospitalization a few days prior to administration and continued hospitalization beyond 2 days after administration is acceptable. 2: Conducted prior to the administration of ADR-001 on the day before or on the day of administration. 3: Collect between 2 days prior to dosing and administration of ADR-001. 4: One day after the second dose is the day of the second dose + one day (0 days allowed). 5: Two days after the second dose is the day of the second dose + 2 days (0 days allowed). 6: Discharge after confirming the coagulation test results.
