# Supplementary figures and images for: Protocol for a Phase 1, Open-Label, Multiple-Center, Dose-Escalation Study to Evaluate the Safety and Tolerability of ADR-001 in the Treatment of Immunoglobulin A Nephropathy
Source: Front Med (Lausanne). 2022 May 27;9:883168. doi: 10.3389/fmed.2022.883168 (PMC9186503; doi:10.3389/fmed.2022.883168)

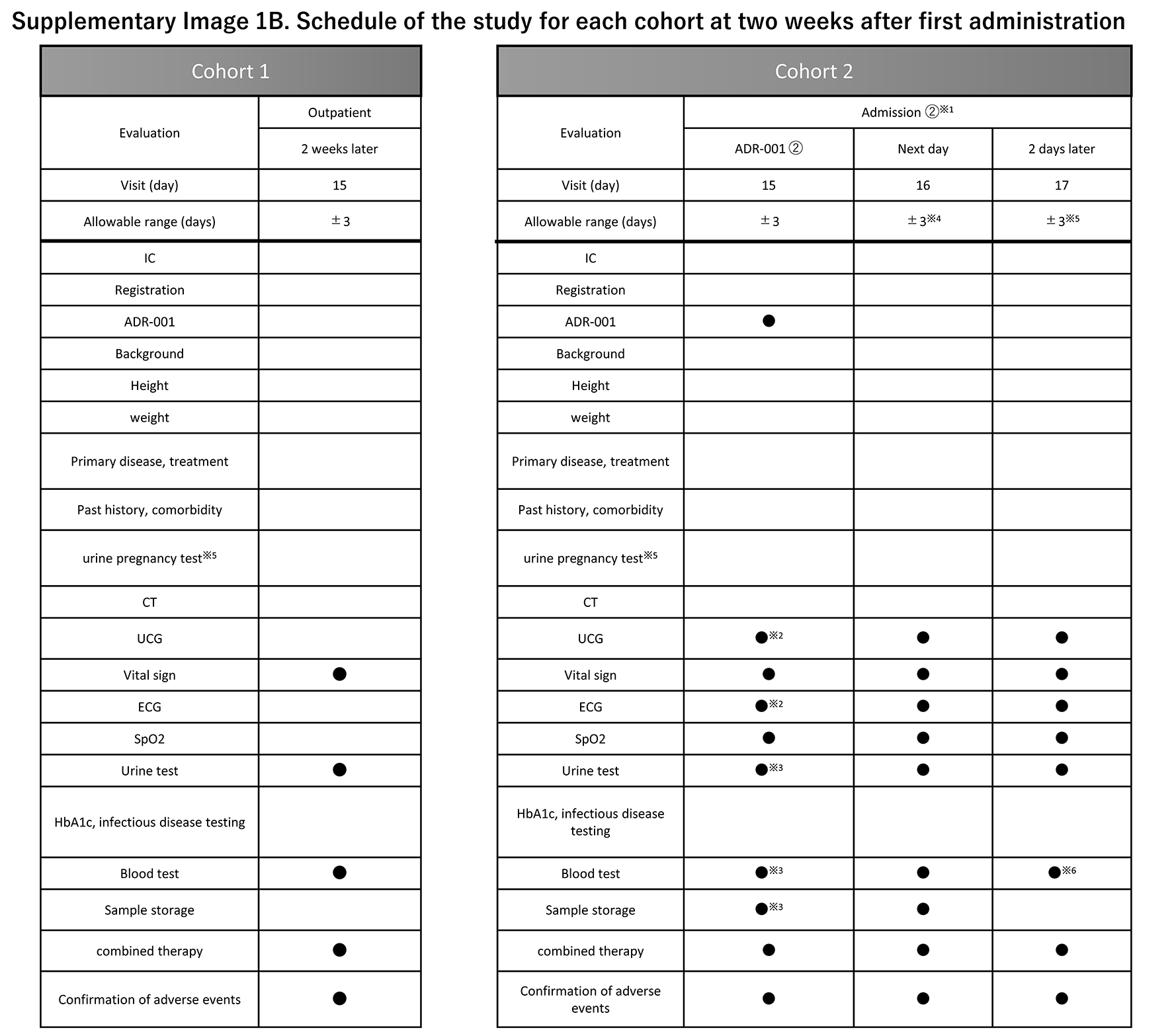

Supplement: Supplementary file 1 [file Image_2.tif]
